# The role of ruminant urine and faeces in the recycling of nutrients by forages

**DOI:** 10.1038/s41598-024-66648-z

**Published:** 2024-07-11

**Authors:** Pei-Tzu Kao, Steve P. McGrath, Heather L. Buss, Tegan Darch, Helen E. Warren, Graham A. McAuliffe, Laura Cardenas, Martin Blackwell, Michael R. F. Lee

**Affiliations:** 1 National Chung Hsing University, Taichung City, 402 Taiwan; 2 Rothamsted Research, North Wyke, Okehampton, Devon EX20 2SB UK; 3 Rothamsted Research, Harpenden, Hertfordshire, AL5 2JQ UK; 4 School of Earth Sciences, University of Bristol, Bristol, BS8 1RJ UK; 5 Alltech Bioscience Centre, Sarney, Summerhill Road, Dunboyne, Co. Meath Ireland; 6 Harper Adams University, Newport, Shropshire, TF10 8NB UK

**Keywords:** Manure, Micronutrients, Sustainable agriculture, Synergism, Antagonism, Dilution, Element cycles, Geochemistry

## Abstract

This study addresses the effect of using animal excreta on the nutritional content of forages, focusing on macro- and micro-element concentrations (nitrogen; N, phosphorus; P, sulphur; S, copper; Cu, zinc; Zn, manganese; Mn, selenium; Se) from animal feed to excreta, soil, and plants. Data were collected from pot and field trials using separate applications of sheep *or* cattle urine and faeces. Key findings indicate that soil organic carbon (SOC) and the type of excreta significantly influences nutrient uptake by forages, with varied responses among the seven elements defined above. Although urine contributes fewer micronutrients compared to faeces (as applied at a natural volume/mass basis, respectively), it notably improves forage yield and micronutrient accumulation, thus potentially delivering positive consequences at the farm level regarding economic performance and soil fertility when swards upon clayey soil types receive said urine in temperate agro-climatic regions (i.e., South West England in the current context). In contrast, faeces application in isolation hinders Se and Mn uptake, once again potentially delivering unintended consequences such as micronutrient deficiencies in areas of high faeces deposition. As it is unlikely that (b)ovine grazing fields will receive either urine or faeces in isolation, we also explored combined applications of both excreta types which demonstrates synergistic effects on N, Cu, and Zn uptake, with either synergistic or dilution effects being observed for P and S, depending largely on SOC levels. Additionally, interactions between excreta types can result in dilution or antagonistic effects on Mn and Se uptake. Notably, high SOC combined with faeces reduces Mn and Se in forages, raising concerns for grazed ruminant systems under certain biotic situations, e.g., due to insufficient soil Se levels typically observed in UK pastures for livestock growth. These findings underscore the importance of considering SOC *and* excreta nutritional composition when designing forage management to optimize nutrient uptake. It should be noted that these findings have potential ramifications for broader studies of sustainable agriculture through system-scale analyses, as the granularity of results reported herein elucidate gaps in knowledge which could affect, both positively and negatively, the interpretation of model-based environmental impact assessments of cattle and sheep production (e.g., in the case of increased yields [beneficial] or the requirement of additional synthetic supplementation [detrimental]).

## Introduction

Forage, including grasses and legumes, is the dominant feed source for ruminants globally^1^. The bioavailability of nutrients in soils greatly affects the yield and nutritional quality of such forages. Soil properties and animal manure, important sources of soil fertilizers, are both important factors affecting the bioavailability of nutrients to forages^2^. However, in a recent review of published data, Kao et al. (2020) demonstrated that studies of nutrient management and cycling in pasture systems have focused primarily on inorganic fertilizers and macronutrients, particularly nitrogen (N) and phosphorus (P)^3–5^. Furthermore, research exploring the impact of organic fertilizers on grass nutrition has mainly concentrated on N^6–8^. Radujković et al.^9^ highlighted a potential oversight in the importance of micronutrients in pasture/grassland production, noting that some soil micronutrients, along with soil physicochemical properties and C:N ratios, are stronger predictors of forage yield than meteorological data and N deposition alone, based on a theory-driven structural equation model .

Forage, as a dominant source of feed for ruminants, must provide sufficient micro-elements, including iron (Fe), manganese (Mn), copper (Cu), zinc (Zn), and selenium (Se) to remain animal health and productivity^2^. Among these micronutrients, Co, Se and I are not classified as essential plant elements. Additionally, elements that primarily occur in cationic forms, such as Zn, Cu, and Mn exhibit different biogeochemical characteristics compared to those in anionic forms such as Se^2,10^. Although animal manures contain sufficient micronutrients and can supply the necessary nutrients for crops^11^, it remains unclear whether applying animal manures effectively improve the status of *all* animal-required nutrients in forages. The roles of manure types and soil properties in this process thus remain a critical knowledge gap^10^.

This study analyzed data from pot and field trials to investigate the impact of applying different types of ruminant excreta (urine and/or faeces) on the status of animal-essential micronutrients (Cu, Zn, Mn, Se) in forages. These four micronutrients were chosen due to their frequent supplementation to ruminants to prevent potential dietary deficiency; from a broader system-scale perspective, the production of synthetic supplements may either improve animal productivity or, in the case of reduced usage via forage-only feed baskets, this could generate fewer impacts to nature by contributing to (semi)circular microeconomies. The main research goals of this study, which indirectly provide novel evidence to conduct cutting-edge sustainability assessments as just alluded to, are to test (1) whether the concentrations of macro- and micro-elements in the urine and faeces of ruminants are affected by the chemical form (organic or inorganic) of minerals supplemented to sheep (tested with the pot trials) or by the composition of the forages of a grazing pasture that maintained cattle (tested with field trials); (2) whether applying urine and/or faeces can effectively improve both yield and the nutritional status of both macro- and micro-elements considering element types, soil organic carbon (SOC) content and manure sources (ruminant species), thereby providing critical economic activity data which underpins scientifically-robust environmental impact assessments as mentioned earlier in this paragraph.

## Materials and methods

### Experimental designs

#### Pot trial

The trial was carried out in a controlled environment with the room temperature maintained at 20 °C and 16 °C during the day (16 h, or ‘h’) and night (8 h), respectively. Light was provided by an artificial light emitting diode (LED) system (light illuminance = 33,000–57,000 lx). Each pot (diameter = 13 cm; height = 20 cm) contained 2.8 kg of soil and was separated into two layers (10 cm each). The top 10 cm layer was either untreated or treated with 70 mL urine and/or 100 g faeces (ca. 22–26 g-dry matter, DM) that were collected from sheep supplemented with either organic or inorganic mineral supplements. Two soils of the same soil type but with different SOC contents (Table 1) were used in the experiment. The organic minerals supplemented to the animals were selenized yeast (Selplex®) and Cu-, Zn-, and Mn-chelate of protein hydrolysate (Bioplex®). The supplemented inorganic minerals were sodium selenite, copper sulfate pentahydrate, oxide, and Mn-oxide. In total, 0.37 mg-Se, 10.1 mg-Cu, 63.5 mg-Zn, and 36.5 mg-Mn were provided to each animal per day for 14 days in total. The doses provided are typically adopted by European industries during ‘in-house’ studies based on the regulation of the National Research Council of the U.S.A^12^. More in-depth details surrounding sheep management can be found in Kao et al.^13^. The pot trial comprised 14 treatment combinations, with four replicates each. Perennial ryegrass (*Lolium perenne* cv. Aber Magic; 0.5 g seed per pot) was sown to achieve three cutting events, with an interval of two weeks between each cut. The experiment followed a Randomized Complete Block Design, with each block containing one replicate of each treatment combination. Samples of forages and leachate were collected at time points as shown in Figure S1.

**Table 1:** Soil properties of the pot and the field trials.

Soil properties		Soils of the pot trial*		Soils of the fields in the field trial			Analytical method and/or instrument
		Soil of lower carbon content	Soil of high carbon content	Permanent pasture dominantly composed of perennial ryegrass (PP)	White clover and perennial ryegrass mix (WC)	Monoculture of high sugar variety of perennial ryegrass (GM)	
Field names		Great Harpenden	Weighbridge Piece	Orchard Dean South	Higher Wyke Moor	Poor Field	–
Soil classification		Batcombe Series		Halstow series	Hallsworth series	Hallsworth series	–
		(Clayden & Hollies, 1984)		British soil classification (Mückenhausen, 1981)			
Soil textural class		Clay loam	Silt clay loam	Clay	Clay	Clay	Pipette by Sedimentation
Cation exchange capacity (cmol kg^−1^)		11.0 ± 0.82	15.4 ± 0.34	14.0 ± 0.45	8.87 ± 0.658	10.5 ± 0.84	Cobalt Hexamine extraction
Soil pH		6.38 ± 0.012	6.31 ± 0.016	5.89 ± 0.018	5.81 ± 0.032	5.96 ± 0.065	10 g soil extracted using 25 mL Milli-Q water for 30 min
Active oxides (mg kg^−1^)	Al	1099 ± 8.9	1087 ± 6.7	1490 ± 114.9	1844 ± 75.8	1466 ± 69.7	Extraction using 0.114 M ammonium oxalate and 0.086 M oxalic acid (Schwertmann, 1964; Rayment and Lyons, 2011; Sparks et al., 2020)
	Fe	4528 ± 56.6	8200 ± 48.7	9951 ± 516.6	7976 ± 238.6	6978 ± 386.5	
	Mn	1506 ± 25.9	1436 ± 23.0	340 ± 43.7	233 ± 39.2	402 ± 175.4	
	P	360 ± 7.4	1003 ± 8.7	702 ± 78.5	527 ± 26.6	411 ± 33.4	
Organic carbon (g kg^−1^)*		15.6 ± 0.39	35.6 ± 0.24	59.5	38.7	38.8	Elemental analyser (NA-1500, Carlo-Erba®)
Total nitrogen (g kg^−1^)		0.02 ± 0.001	0.03 ± 0.001	6.2	4.0	4.1	
2 M KCl extractible N (mg-N/kg-DM soil)		8.7 ± 0.75	15.9 ± 2.82	–	–	–	Photometric analyzer (Aquakem 250, Thermo Scientific®)
Total P (g kg^−1^)		0.81 ± 0.013	1.67 ± 0.058	1.42 ± 0.117	1.05 ± 0.028	1.02 ± 0.052	Acid digestion using aqua regia and analysed with ICP-MS or ICP-OES
Total Fe (g kg^−1^)		33.6 ± 0.61	27.3 ± 0.89	30.8 ± 1.32	32.9 ± 0.98	36.2 ± 5.55	
Total Mn (g kg^−1^)		1.79 ± 0.060	1.61 ± 0.058	0.47 ± 0.068	0.35 ± 0.052	0.58 ± 0.258	
Total Cu (mg kg^−1^)		17.7 ± 0.22	24.1 ± 0.93	23.6 ± 1.40	21.5 ± 3.19	17.9 ± 2.18	
Total Zn (mg kg^−1^)		72.1 ± 2.22	101 ± 3.4	77.8 ± 3.51	65.4 ± 1.23	80.5 ± 3.95	
Total Se (μg kg^−1^)		782 ± 14.0	865 ± 26.6	841 ± 110.4	1014 ± 5.5	1312 ± 50.7	
Extractable Se (μg kg^−1^)		9.39 ± 0.101	9.30 ± 0.102	35.0 ± 2.57	43.1 ± 1.52	50.0 ± 1.11	ICP-MS analysis following extraction (5 g soil in 25 mL 0.016 M KH_2_PO_4_)

*Soil property data for the pot trial from Kao et al.^13^.

#### Field trial

Three cattle-grazing pasture fields trialed on the North Wyke Farm Platform (NWFP; 50°45′N, 3°50′W, http://nwfp.rothamsted.ac.uk), each under different pasture management strategies, were selected due to their equal soil types, topographical nature, and areas. Urine and faeces collected from cattle within each field were applied back to the same fields thus ensuring that soil-feed-nutrition uniqueness across treatments was maintained. The three pasture management strategies were:

#### Permanent pasture (PP)

The fields were predominantly composed of perennial ryegrass (*Lolium perenne*). All PP land utilized had not been ploughed for at least 20 years. The fields received regular application of inorganic N fertilizer at a standard rate (40 kg N * 3 = 120 kg N total, as ammonium nitrate, throughout spring and summer as detailed in McAuliffe et al.)^14^; (2) White clover (*Trifolium repens*) and perennial ryegrass (*Lolium perenne* cv. AberMagic) mix (WC): the fields were converted from PP in July 2013 through soil inversion cultivation with a target to achieve 30% white clover ground cover. No inorganic N fertilizer was applied in most of the years in the fields with WC (including the year the present experiment was conducted); the only notable exception was that a minor amount of N was applied at the start of trial conversion when it was needed prior to satisfactory legume establishment. It should be noted that no N fertilizer was applied to WC during the 12 months prior to experimental commencement, either; (3) Perennial ryegrass monoculture (GM): the fields were converted from PP in July 2014, also through soil inversion cultivation, and were managed in the same way as the fields of PP described above with one important difference; namely, the same perennial ryegrass in WC (cv. AberMagic) was used as a monoculture.. More details about the background of the study site, including cattle management, climate, rainfall and the setup of NWFP, can be found in McAuliffe et al.^14^. The soil properties of the three fields are shown in Table 1. Three experimental blocks, each 50 m apart from each other in a triangular shape near the field entrance, were established in each of the three fields. Within each of the experimental block, small plots (2.5 m × 1.5 m) were established and amended with either collected cattle urine, collected cattle faeces, or a control with no excreta. Figure S2 shows the timeline of the field trial and the management of the field before the start of the experiment. Experimental excreta were applied on the 6th June in 2017, when the rainfall in the 30 days following treatment application was 55 mm (a dry period relative to rolling monthly averages in the region). Forage samples were collected on 10th August in 2017 and were stored and processed ready for application as detailed in the next section.

### Collection, management and application of the urine and faeces

#### Pot trial

Urine and faeces were collected separately from 12 male Charolais × Suffolk-Mule sheep yearlings at 12–18 months of age (mean weight = 57 ± 2.9 kg, Body Condition Score = 3.3 ± 0.20). Six animals were given organic mineral supplements and the other six animals were given inorganic mineral supplements. Each sheep was penned individually with access to an individual feeding bin of which the weight of feed was automatically measured and recorded (Figure S3a). The separate faeces and the urine were collected on a daily basis during the supplementation period (14 d) using the facility as shown in Figure S3b. Urine and faeces applied to soils were collected on the 14th day of the supplementary period, with the urine and faeces from the six animals mixed and homogenized before being applied to the soils. The application amount of the urine was calculated based on the average surface area of a urine patch (290 cm^2^) and the average volume of each urination (150 mL) reported by Doak^15^, which gave a urine density of 0.52 mL cm^−2^ (ca. 70 mL per pot). The daily excretion ratio of urine and faeces from 24 sheep across two weeks in a previous sheep experiment^13^ was on average 4.764 (mL urine/g-DM faeces). The amount of faeces applied was between 22 and 26 g-DM per pot due to the variation in moisture of the faeces.

#### Field trial

Within the two months prior to the first treatment application day, the experimental urine and faeces were collected from cattle that grazed in each respective field, as briefly mentioned above. The urine was collected on two dates (22nd April and 2nd May, 2017) during natural urination events in a cattle handling facility. After collection, the urine was kept in a freezer at − 20 °C until three days before application, when it was gradually defrosted at 4 °C and subsequently homogenized in bulked barrels for PP, WC and GM, separately. The urine was applied at a rate of 5 L m^−2^^16^. Faeces were collected on many trips from the fields from freshly voided cowpats using a ladle and homogenized in barrels and stored at 4 °C until the day of application. The faecal application rate was 20 kg m^−2^^17^ in the designated area (2 * 1 m^2^), spread evenly as is standard in similar studies.

### Collection and management of soil samples

Soils used in the pot trial were air-dried and sieved (< 2 mm) using a stainless-steel mesh prior to chemical analysis. Representative soils (at 0–10 cm depth) of the field trial were sampled on 2nd June 2017 and 2nd August 2017, and freeze-dried prior to analysis. The methods and the instruments used to analyze the soil properties of the pot and the field trials are provided in Table 1.

### Collection and management of forage samples

In the pot trial, grass grown in each pot was cut at 2 cm above the soil surface using scissors with stainless-steel blades and stored at − 20 °C before freeze-drying. The DM yield of the grass sampled from each pot was determined by measuring the difference in weight before and after freeze-drying. In the field trial, forage sampled at 23rd May 2017 and 10th August 2017 from a specific designated area for the current study (50 cm × 50 cm) in each plot was oven dried at 85 °C for 24 h for the determination of DM yield.

### Sample analysis

#### Total element analysis using ICP-OES and ICP-MS

Prior to analysis, the faecal samples were oven-dried at 80 °C for three days, finely ground (using a coffee grinder (BR-CG3-UK, Brewberry®)), and acid-digested using a microwave digestion system (0.25 g sample digested for 60 min in 3 mL concentrated HNO_3_ followed by the digestion in 3 mL Milli-Q water (18 MΩ) and 2 mL H_2_O_2_ at 115 °C for 1 min followed again by digestion at 175 °C for 10 min). The urine samples were filtered with a 0.45 μm syringe filter, and diluted 20-fold to a final concentration of 5%(v/v) HNO_3_ and 1%(v/v) methanol. The soil samples were finely ground using a ball mill and acid-digested (0.25 g sample in 5 mL aqua regia). The forage samples were freeze-dried and finely ground following the same procedure of the faecal samples. All the prepared analytes for total element analysis (P, S, Cu, Zn, Mn and Se) were analysed at the Analytical Chemistry Unit (ACU) of Rothamsted Research using ICP-OES (Perkin Elmer® Optima™ 7300DV and Agilent® 5900 SVDV) or ICP-MS (Perkin Elmer® NexION 300X). For each element, analyte concentrations greater than 50 μg L^−1^ were analyzed using ICP-OES, and by ICP-MS for concentrations below50 μg L^−1^. The ICP-OES settings were: sample uptake = 1 mL min^−1^; nebulizer gas flow = 0.7 L min^−1^; auxiliary gas flow = 0.3 L min^−1^; plasma flow = 17 L min^−1^; RF power = 1400 Watts. The ICP-MS settings were: sample loop size = 1 mL; nebulizer gas flow = 0.91 L min^−1^; auxiliary gas flow = 1.2 L min^-1^; plasma flow = 18 L min^−1^; radio frequency (RF) power = 1600 Watts, kinetic energy discrimination (KED) mode at 3 mL min^−1^ He. The isotope mass and wavelength used and the estimated detection limit of each element in the ICP-OES and ICP-MS are shown in Table S1.

#### Total N analysis

The TN concentration in the urine samples were determined using a photometric analyzer (Aquakem 250, Thermo Scientific®). The urine samples were kept in a freezer at − 20 °C and defrosted at the day of analysis. On the day of analysis, each sample was filtered through a 0.45 μm Nylon syringe and diluted 50 times with Milli-Q water (18 MΩ) to make the final analyte. The concentrations of TN in the analytes of soil and faeces were determined using an elemental analyser (NA-1500, Carlo-Erba®). The sample preparation method was the same as that of ICP analyses.

### Statistical analysis and calculation

**Figure 1:**
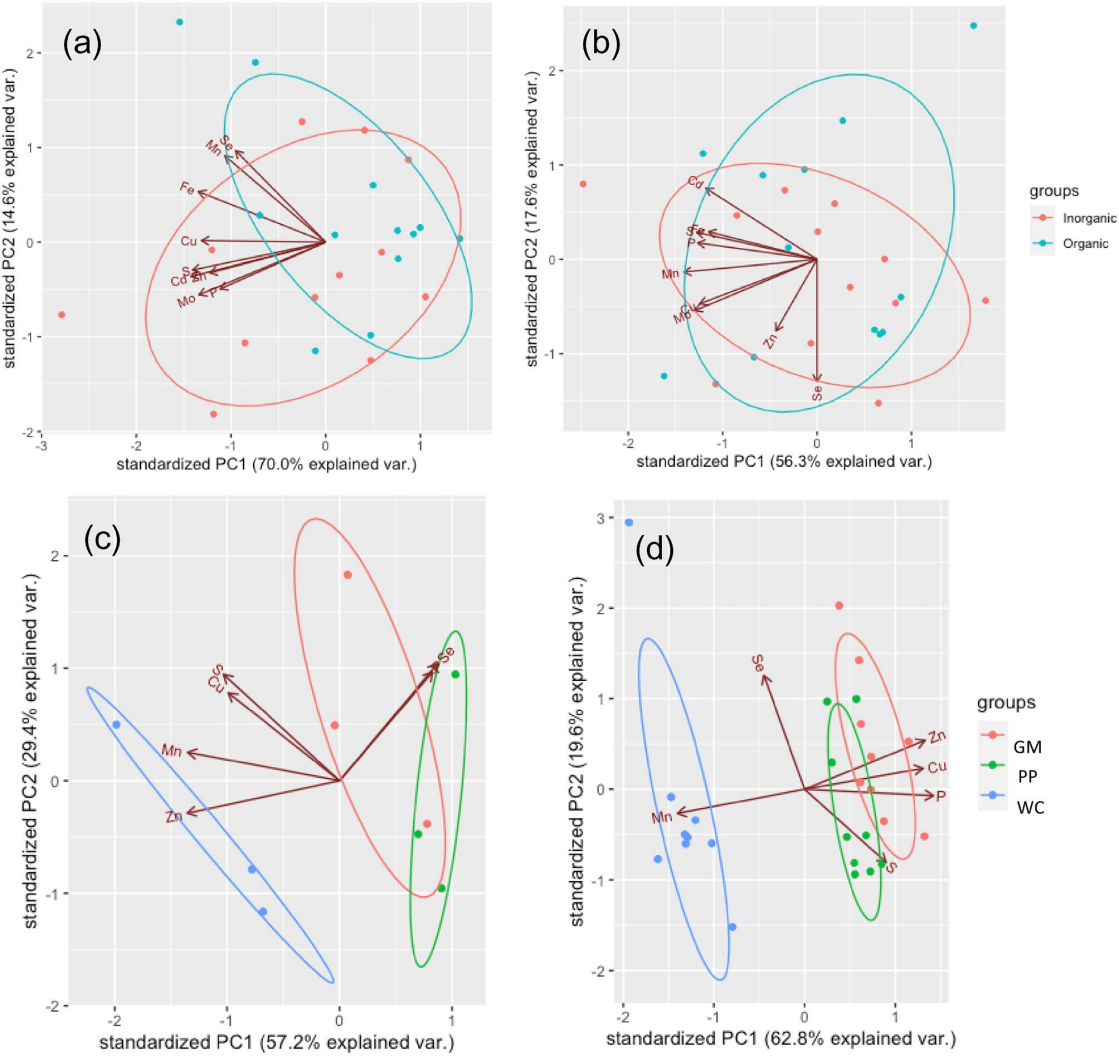
PCA groupings based on the nutrient concentrations in the urine or faeces from different sources. (**a**) urine and (**b**) faeces of the sheep given different chemical forms (inorganic and organic) of mineral nutrient supplements; (**c**) urine and (**d**) faeces of the cattle grazing in fields of different farm management practices (GM, PP and WC).

**Figure 2:**
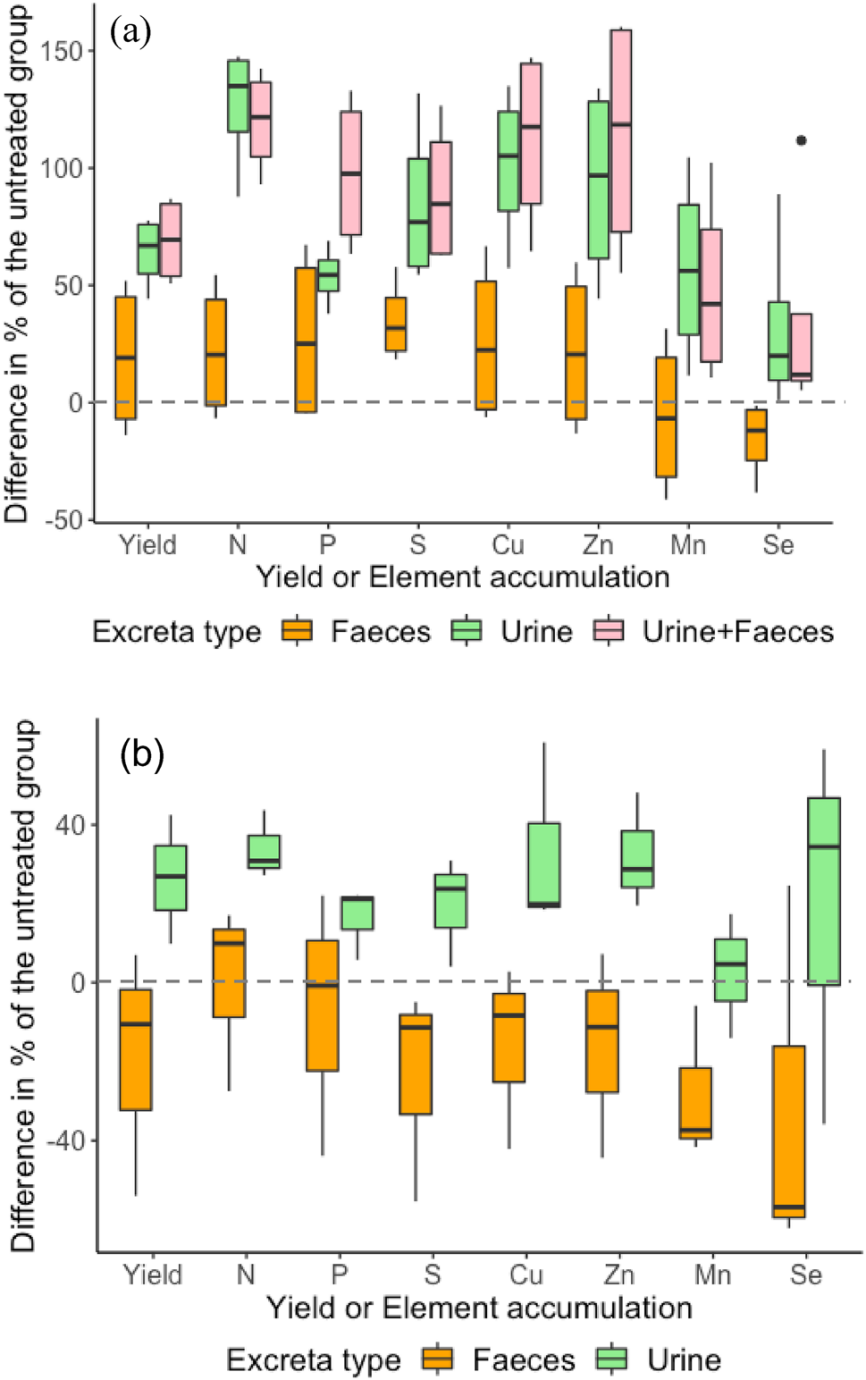
The difference (%) relative to the untreated groups in the yield or elemental accumulation in the forages of the different excreta treatment groups for (**a**) the result of the perennial ryegrass in the pot trial and (**b**) the result of the forages of different pasture management practices in the field trial.

**Figure 3:**
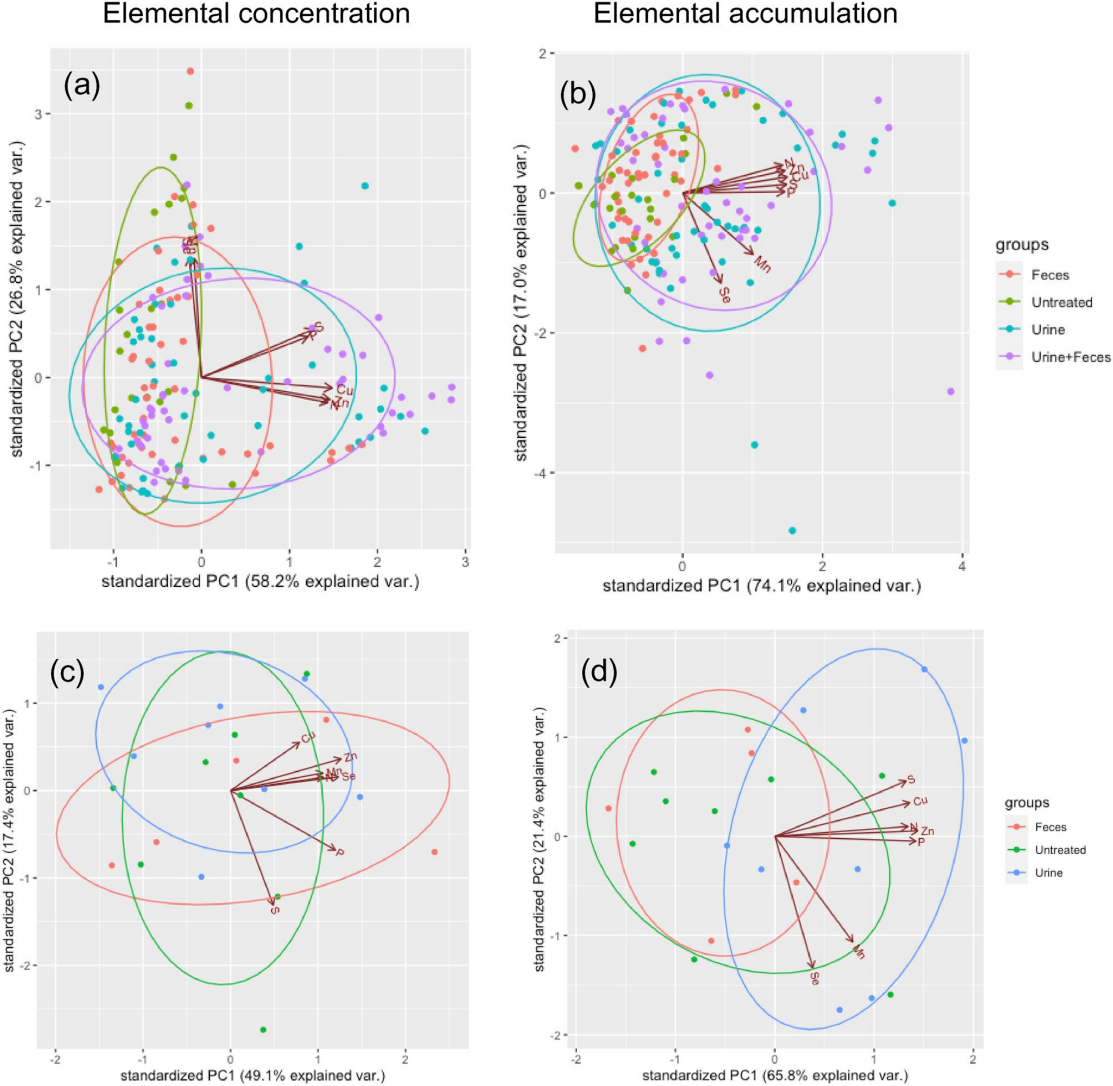
PCA results of forages grouped by the type of excreta applied according to (**a**) elemental concentrations in the forages of the pot trial, (**b**) elemental accumulations in the forages of the pot trial, (**c**) elemental concentrations in the forages of the field trial, and (**d**) elemental accumulations in the forages of the field trials.

All the statistical analyses were performed in R software (v.4.3.1) (URL:https://www.R-project.org/)^18^. The normal distribution of the data for the following statistical analyses were checked using Q–Q plots. ANOVA models (y ~ block + the form of the given mineral supplement), and (y ~ field) were used to analyze the impact of different treatments for sheep and cattle, respectively, on the nutrient composition in their urine and faeces (Table 2). A factorial ANOVA model (y ~ block + soil + excreta + soil*excreta) was used to analyze the impact of the excreta and soil on DM yield and the accumulation of nutrients in the perennial ryegrass of the pot trial (Table 3). In the field trial, the urine and faeces collected from different fields were not mixed together before re-applying back to the individual field, therefore we were not able to separate the impact of the applied urine and faeces on forages of different field sites from location variation. Therefore, an alternative ANOVA model (y ~ excreta) was used to analyse the elemental accumulation in the forages collected from each field individually (Table 4). If significant differences (*P* < 0.05) were identified, post hoc comparisons of Fisher’s LSD (α = 0.05) were performed. The PCA analyses of Figs. 1 and 3 were performed using the elemental concentrations in the urine and faeces of different treatments, and the elemental concentrations and accumulations in the forages of different treatments, respectively. The percentage difference in yield and total elemental accumulation in forages relative to the untreated groups (Fig. 2) was calculated using Eq. 1.

**Table 2:** Nutrient concentrations in the urine and feces of sheep and cattle given different treatments.

Excreta type	Element	Excreta collected from sheep given^1^*		*P*-value	Excreta collected from cow grazing at farmlet^2^			*P*-value
		Organic mineral supplements (mean ± SEM, *n* = 12)	Inorganic mineral supplements (mean ± SEM, *n* = 12)		PP (mean ± SEM, *n* = 9 for feces, *n* = 3 for urine)	WC (mean ± SEM, *n* = 9 for feces, *n* = 3 for urine)	GM (mean ± SEM, *n* = 9 for feces, *n* = 3 for urine)	
Faeces	N (g kg^−1^)	N.A	N.A	N.A	31.7 ± 0.31^c^	34.6 ± 0.37^a^	33.6 ± 0.23^b^	< 0.001***
	P (g kg^−1^)	12.6 ± 0.32	12.9 ± 0.48	0.5271	10.1 ± 0.06^a^	9.48 ± 0.054^b^	10.1 ± 0.06^a^	< 0.001***
	S (g kg^−1^)	3.72 ± 0.063	3.68 ± 0.102	0.7995	3.56 ± 0.011	3.53 ± 0.023	3.59 ± 0.009	0.057
	Cu (mg kg^−1^)	46.7 ± 1.95	48.9 ± 1.14	0.3150	28.0 ± 0.09^a^	23.2 ± 0.25^c^	26.6 ± 0.13^b^	< 0.001***
	Zn (mg kg^−1^)	323 ± 16.5	336 ± 24.7	0.6119	80.9 ± 0.42^b^	73.4 ± 0.57^c^	84.7 ± 0.65^a^	< 0.001***
	Mn (mg kg^−1^)	416 ± 10.9	411 ± 13.2	0.7853	500 ± 2.4^b^	667 ± 7.0^a^	423 ± 4.0^c^	< 0.001***
	Se (μg kg^−1^)	467 ± 44.1	417 ± 34.1	0.9472	172 ± 12.1^b^	212 ± 12.0^a^	214 ± 13.2^a^	0.040*
Urine	N (g L^−1^)	6.66 ± 1.169	7.63 ± 1.400	0.4110	3.31 ± 0.032^a^	1.96 ± 0.034^b^	1.73 ± 0.020^c^	< 0.001***
	P (mg L^−1^)	2.91 ± 0.618	4.76 ± 0.882	0.1143	14.7 ± 1.25	13.0 ± 0.35	15.5 ± 0.86	0.207
	S (g L^−1^)	1.06 ± 0.103	1.28 ± 0.151	0.1952	0.325 ± 0.018	0.388 ± 0.0398	0.383 ± 0.0291	0.310
	Cu (μg L^−1^)	40.3 ± 3.91	48.2 ± 9.98	0.4413	14.3 ± 2.54	24.1 ± 3.26	21.0 ± 7.56	0.414
	Zn (mg L^−1^)	5.15 ± 0.994	4.98 ± 0.703	0.8704	0.172 ± 0.0130^b^	0.706 ± 0.0750^a^	0.239 ± 0.0153^b^	< 0.001***
	Mn (μg L^−1^)	115 ± 21.0	122 ± 16.3	0.7434	31.4 ± 1.24^b^	52.9 ± 6.13^a^	39.8 ± 2.63^ab^	0.022*
	Se (μg L^−1^)	19.3 ± 2.35	25.4 ± 3.76	0.1272	78.5 ± 6.11^a^	53.9 ± 5.82^b^	76.8 ± 4.84^a^	0.037*

N.A.: Not analysed. Symbols ‘*’, ‘**’, ‘***’ indicate statistical significances of ANOVA test (^1^y ~ block + the form of the mineral supplement; ^2^y ~ field) at p-value < 0.05, < 0.01, < 0.001, respectively. The lowercase English letters in the same row represent the statistical results of post-hoc LSD test, after a significant result of the ANOVA test. *In total 0.37 mg-Se, 10.1 mg-Cu, 63.5 mg-Zn, and 36.5 mg-Mn were provided to each animal per day for in total 14 days.

**Table 3:** Element accumulation in the perennial ryegrass in the pot trial.

Different soils	Excreta type	Yield (g per pot)	N (mg)	P (mg)	S (mg)	Cu (ug)	Zn (ug)	Mn	Se (μg)
Soil of low organic matter content	Untreated (*n* = 4)	4.09 ± 0.164^c^	6.29 ± 0.18^c^	12.5 ± 0.83^c^	8.35 ± 0.417^b^	21.7 ± 0.96^c^	68.4 ± 5.48^c^	357 ± 20.0^b^	0.19 ± 0.012^a^
	Treated with urine (*n* = 8)	7.21 ± 0.233^a^	15.5 ± 0.55^a^	19.2 ± 0.86^b^	13.1 ± 0.39^a^	49.1 ± 1.92^a^	157 ± 5.8^a^	676 ± 39.7^a^	0.22 ± 0.043^a^
	Treated with faeces (*n* = 8)	6.02 ± 0.287^b^	9.27 ± 0.35^b^	20.0 ± 0.85^b^	12.4 ± 0.58^a^	33.9 ± 1.35^b^	105 ± 4.1^b^	441 ± 41.5^b^	0.18 ± 0.023^a^
	Treated with urine and faeces (*n* = 8)	7.58 ± 0.301^a^	15.0 ± 0.73^a^	28.3 ± 1.27^a^	13.6 ± 0.53^a^	53.1 ± 2.43^a^	177 ± 10.0^a^	655 ± 38.8^a^	0.20 ± 0.011^a^
Soil of high organic matter content	Untreated (*n* = 4)	8.45 ± 0.670^B^	14.0 ± 1.43^B^	24.6 ± 2.22^B^	10.7 ± 1.14^B^	49.3 ± 4.40^B^	202 ± 21.0^B^	612 ± 50.2^B^	0.11 ± 0.010^AB^
	Treated with urine (*n* = 8)	12.8 ± 0.306^A^	28.9 ± 1.03^A^	37.7 ± 1.66^A^	22.8 ± 0.85^A^	85.6 ± 3.83^A^	314 ± 13.1^A^	754 ± 38.4^A^	0.16 ± 0.024^A^
	Treated with faeces (*n* = 8)	7.67 ± 0.539^B^	13.6 ± 1.00^B^	23.5 ± 1.48^B^	13.0 ± 0.54^B^	47.3 ± 3.26^B^	184 ± 14.4^B^	399 ± 25.9^C^	0.08 ± 0.010^B^
	Treated with urine and faeces (*n* = 8)	12.9 ± 0.469^A^	28.2 ± 0.87^A^	41.5 ± 1.24^A^	23.2 ± 0.77^A^	87.7 ± 3.86^A^	337 ± 15.2^A^	705 ± 40.4^AB^	0.18 ± 0.034^A^
*P*-values of the ANOVA test	Excreta effect	< 0.001***	< 0.001***	< 0.001***	< 0.001***	< 0.001***	< 0.001***	< 0.001***	0.038*
	Soil effect	< 0.001***	< 0.001***	< 0.001***	< 0.001***	< 0.001***	< 0.001***	0.028*	< 0.001***
	Interaction	< 0.001***	< 0.001***	< 0.001***	< 0.001***	< 0.001***	0.005**	0.007**	0.407

Symbols ‘*’, ‘**’, ‘***’ indicate statistical significances of ANOVA test (y ~ block + soil + excreta + soil*excreta) at *p*-value < 0.05, < 0.01, < 0.001, respectively. Denoted significant interaction effect between the excreta type and soil in the ANOVA tests, the effect of excreta type applied in the same soil was further compared. The lowercase and the uppercase English letters in the same column represent the results of post-hoc LSD test of the treatments in the low and high organic matter soil, respectively.

**Table 4:** Element accumulation in the forages in the field trial.

Different fields	Treatment of animal excreta	Yield (g per cut)	N (g)^‡^	P (mg)	S (mg)	Cu (μg)	Zn (mg)	Mn (mg)	Se (μg)
PP	Untreated (*n* = 3)	109 ± 15.9^a^	249 ± 55.8 (*n* = 2)	309 ± 37.6^a^	237 ± 52.9	733 ± 149.1	2.66 ± 0.686	9.00 ± 2.159	5.56 ± 5.022
	Treated with urine (*n* = 3)	120 ± 2.9^a^	317 ± 19.0(*n* = 2)	327 ± 11.2^a^	247 ± 13.1	878 ± 80.3	3.18 ± 0.157	10.6 ± 4.09	9.45 ± 3.820
	Treated with faeces (*n* = 2)	50.2 ± 9.29^b^	181 (*n* = 1)	174 ± 19.5^b^	106 ± 29.2	424 ± 107.1	1.48 ± 0.289	5.25 ± 0.465	3.03 ± 0.008
Excreta effect (*p*-values)		0.0211*	-	0.0278*	0.1080	0.1273	0.1535	0.5541	0.6075
WC	Untreated (*n* = 3)	91.9 ± 20.08	219 ± 58.8	308 ± 45.9	227 ± 26.2	720 ± 180.0	2.30 ± 0.598	13.1 ± 5.90	6.93 ± 1.987
	Treated with urine (*n* = 3)	117 ± 7.5	287 ± 24.0	376 ± 36.8	281 ± 13.3	853 ± 118.6	2.96 ± 0.345	13.7 ± 3.36	11.0 ± 1.874
	Treated with faeces (*n* = 3)	98.4 ± 13.03	241 ± 41.5 (n = 2)	376 ± 25.1	216 ± 17.2	740 ± 78.3	2.46 ± 0.134	12.3 ± 0.63	8.63 ± 0.868
Excreta effect (*p*-values)		0.4978	0.5664	0.3828	0.1173	0.7555	0.5267	0.9699	0.2876
GM	Untreated (*n* = 3)	131 ± 20.1^a^	228 ± 58.8	351 ± 40.0	298 ± 45.4^ab^	867 ± 116.5^b^	2.87 ± 0.279^b^	14.5 ± 4.10	2.71 ± 0.848
	Treated with urine (*n* = 3)	187 ± 8.5^a^	328 ± 24.0	425 ± 17.4	390 ± 2.7^a^	1394 ± 165.3^a^	4.26 ± 0.211^a^	12.4 ± 1.28	1.74 ± 1.132
	Treated with faeces (*n* = 3)	117 ± 2.6^b^	267 ± 41.5	348 ± 13.3	264 ± 8.9^b^	795 ± 91.1^b^	2.55 ± 0.131^b^	9.05 ± 0.562	1.03 ± 0.575
Excreta effect (*p*-values)		0.0183*	0.0904	0.1425	0.0375*	0.0312*	0.0031**	0.3666	0.4479

Symbols ‘*’, ‘**’ indicate statistical significances of ANOVA test (y ~ excreta) at *p*-value < 0.05, and < 0.01, respectively. ^‡^There were missing datapoints for the results of N in PP and WC. The available sample numbers of the missing datapoints were noted in the parenthesis after the analytical results. The lowercase English letters in the same column represent the result of post-hoc LSD test, after the denoted significance ANOVA test result, between the different treatments in the same field.

1 $$Difference \left(\%\right) relative to the untreated group= \frac{{(Y}_{treated}-{Y}_{untreated})}{({Y}_{untreated})}\times 100\%$$

*Y = the responses of the different treatments, such as grass yield and the elemental accumulation.

### Ethics approval

All procedures (none of which required anaesthesia or euthanasia) were conducted in accordance with the United Kingdom Animal (Scientific Procedures) Act 1986, approved by institutional ethical review committees (Rothamsted Research, Animal Welfare and Ethical Review Board) and conducted under the authority of the Project Licence number P592D2677. The study is reported in accordance with the ARRIVE guidelines (https://arriveguidelines.org). Animals in the sheep and cattle experiment were assessed daily for health and well-being, as determined by alertness, feed and water intake.

## Results

### Nutrient composition in urine and faeces collected from different sources

The concentrations of macro- (P and S) and micro- (Cu, Zn, Mn and Se) elements were not different in the urine nor the faeces collected from sheep given mineral supplements of different chemical forms (organic or inorganic; Table 2). The N concentrations in the urine were not different across the sheep that received different mineral supplements either (Table 2). Similarly, the PCA analysis showed that the nutrient composition of urine and faeces were not distinctly separated by the treatment of different forms of mineral supplements (Fig. 1a, b). In the case of cattle, the concentrations of N, P, Cu, Zn, Mn and Se in the faeces and N, Zn, Mn and Se in the urine collected from cattle were significantly different between the pasture fields (Table 2). The PCA analysis showed that the nutrient composition of both the urine and faeces of WC field was significantly distinct from those of GM and PP (Fig. 1c, d). The cattle faeces of WC had significantly higher concentrations of N, Mn and Se and lower concentrations of Cu and Zn than that of GM and/or PP (Table 2). Cattle urine sourced from WC had significantly higher Zn and Mn, and lower Se, than that of GM and/or PP.

### DM yield and elemental accumulation in forages of different treatments

The results of the pot trial indicate that the yield and the elemental accumulation of forages depended on *both* the soil properties and excreta type, as reflected by the significant interactive effect between the soil and excreta type on the DM yield and the accumulation of N, P, S, Cu, Zn and Mn (Table 3). The accumulation of Se in swards was affected more by soil than by excreta type (Table 3). Relative to the untreated soils, the application of sheep urine, both with or without faeces, significantly increased the DM yield of perennial ryegrass and the accumulation of N, P, S, Cu, Zn and Mn—but not Se—in the pasture (Table 3). The application of faeces lacked a significant effect compared to urine on forage yield and nutrient accumulation, and further, varied with soil (Table 3). Relative to the untreated soils, the application of faeces to the soil with the lowest SOC content resulted in higher grass yield and accumulation of N, P, S, Cu, Zn and Mn, whereas when faeces was applied to the soil with the highest SOC content, no significant increase in these parameters occurred (Table 3).

In the field trial, the application of urine appeared to increase the yield of forages across the three fields relative to the untreated control plots, though urine did not increase to a statistically significant level (Table 4). Similarly, a statistically higher accumulation of nutrients in the forages was only observed in Cu and Zn in the urine-treated plots of the GM field. The application of faeces significantly decreased the yield of forages in the PP and GM fields, which was reflected in the lower accumulation of P in the grass of PP field. Although not statistically significant, the accumulation of S, Cu, Zn, Mn, and Se in the forages of both PP and GM fields treated with faeces appeared to be lower than in the forages of the untreated control plots. The impact of applying excreta on the yield and nutrient accumulation was not significant in the WC field.

In both the pot and field trials, the application of urine appeared to increase the accumulation of all the measured nutrients by plants, regardless of the difference in soils and fields (Fig. 2). The application of faeces, however, decreased the accumulation of Mn and Se by plants in the pot trial and of P, S, Cu, Zn, Mn and Se in the field trial. Notably, the decrease in accumulation of Mn and Se related to the application of faeces was balanced by the concurrent application of urine (Fig. 2a), although the accumulations were still lower than in the urine-only treatments.

### Forages nutritional composition resulted from different treatments

Different excreta type significantly shifted the overall nutritional composition in forages as shown by the PCA analyses of elemental concentrations and accumulation in the forages (Fig. 3). The application of urine, either with or without faeces, shifted the nutrient composition of the forages to a greater extent than faeces (Fig. 3a, b). In contrast, the result of the faecal treatment was largely overlapped with the untreated groups, indicating little impact on altering the nutritional status in forages. The grouping results in the pot trial were more significant than that in the field trial as a result of more datapoints, thus statistical power (Fig. 3). Significant elemental groups can be seen in Fig. 3a, with Cu, Zn, and N as one group, P and S another, and Mn and Se as a third. For elemental accumulation in forages, the two experiments showed comparable results with Mn and Se as a group, and the rest of the elements as another group (Fig. 3b, d).

## Discussion

### The impact of mineral supplementation and pasture management on the nutrient composition of sheep and cattle excreta

The concentrations of nutrients in the excreta was not affected by the chemical forms of mineral supplements given to the sheep (Table 2). However, the nutrient concentrations of the excreta were affected by the different pasture management systems (Table 2). This response most likely relates to the inherent differences across fields as shown by Lee et al.^19^ who reported different concentrations of mineral nutrients in forages across the three management practices on the NWFP. Indeed, the composition of feeds are known to influence the nutrient composition in the animal excreta (urine and/or faeces)^20^, yet to the best of our knowledge this is the first ‘duel’ approach (i.e., pot and plot) to such studies. Herein, the nutrient concentrations in the forages collected from each field prior to excreta collection (i.e. the forages the cattle consumed) indicates a link between likely dietary intake (feed mineral nutrient concentrations) and excretion. For example, the forages (Table S2) and excreta (both urine and faeces; Table 2) collected from the WC field had higher concentrations of Mn than the forages and excreta from the other fields. However, the reason for the higher concentration of Mn in the forages of WC is not clear. We did not control the initial soil nutrient concentrations across the fields so it was not possible to assess whether the differences in WC, PP, and GM excreta nutrient concentrations (Fig. 1c, d) resulted from the different forage species composition, different soil properties, or to add further complexity, the interaction of both. However, the PCA analysis of soil sampled in June 2016, approximately one year before the start of the field trial, shows that the soil of WC was not distinct from the other treatments (Figure S4), which implies that the soil nutrient composition may not be the main cause of the forage nutrient composition difference between fields. Therefore, the high concentration of Mn in the forage of WC likely reflects the forage species composition or field management rather than the difference between the treatment fields. The reported concentrations of Mn in the dominant forage types in the experimental fields differ somewhat, ranging from 22.4 to 98.8 mg kg^−1^ DM in white clover (sown only in WC) and 15–127.2 mg kg^−1^ DM in perennial ryegrass, depending on the cultivars and the experimental conditions^2^. Moreover, the retention and availability of forage Mn varies between perennial ryegrass and white clover^21^. However, we cannot definitively credit the presence of white clover with increased intake of Mn by ruminants. There are few studies in the literature relating forage species compositions and populations to the excretion of both macro- and micro-nutrients by grazing animals, which merits further study. This extant gap in knowledge is becoming increasingly pertinent, particularly as agricultural sustainability is often driven by proxy through crop/livestock productivity, whilst good quality data underpinning sustainability models is scarce^22^ as reinforced herein.

### The impacts of applying ruminant urine and faeces in soils on the nutrient composition of forages

Despite more input of total nutrients from faeces than from urine (Table S3), the nutrient accumulation in the forages did not reflect the input of nutrients from the excreta types applied, contradicting our prediction. The application of urine increased the nutrient accumulation in the forages, whereas the application of faeces had minor or negative impact on the nutrient accumulation (Fig. 2). Urine is a liquid fertilizer and the nutrients contained in urine therefore are more easily accessible to plant roots and are mostly bioavailable; faeces, on the other hand, initially sits on the soil surface and is mainly composed of slow-release chemical forms of nutrients However, a sequential extraction of Cu, Zn and Mn, using the method proposed by Bureau Community of Reference (BCR)^23^, showed that sheep faeces used in the present pot trial (ca. 6 to 7%, 39 to 41%, and 79 to 82% of Cu, Zn and Mn, respectively) were present in the exchangeable fraction, which were likely bioavailable to plants^13^. If only 10% of the nutrients in the applied faeces are bioavailable, the amount of bioavailable nutrients (except for S and Se) contributed from the faeces would still be more than the amount contributed from the urine (Table S3). Furthermore, the concentrations of soil extractable Cu, Zn and Mn in the pot trial were significantly higher in the soils that received faeces compared to the soils that received urine (Table S4), implying that bioavailability of nutrients in the urine and faeces was not the only reason for the higher accumulation of nutrients in the forage grown on urine-treated soils. Soil extractable N and potassium (K) after applying excreta were higher in urine treatment compared with faecal treatment in the present study (Table S4). The major driver of the higher nutrient accumulation in forages of the urine treatment was likely a synergistic effect (discussed in the following section) between N, dominantly excreted via urine, and other elements, whereby the bioavailable N from urine improved the grass yield, which, in turn, increased the uptake of other elements (Table 5).

**Table 5:** Initial indicators of the response of nutrients in forages to the application of excreta in the pot trial.

	Treatment	Forage yield (compared to the controls)	N	P	S	Cu	Zn	Mn	Se
Soil of low organic matter content	Urine	Increase	Synergism	Dilution	Dilution	Synergism	Synergism	Synergism	Dilution
	Faeces	Increase	Synergism	Synergism	Synergism	Synergism	Synergism	Dilution	Dilution
	Urine + Faeces	Increase	Synergism	Synergism	Dilution	Synergism	Synergism	Dilution	Dilution
Soil of high organic matter content	Urine	Increase	Synergism	Synergism	Synergism	Synergism	Synergism	Dilution	No response (change < 1 μg/kg)
	Faeces	Not significantly change	Synergism	Synergism	Synergism	Synergism	Synergism	Antagonism	Antagonism
	Urine + Faeces	Increase	Synergism	Synergism	Synergism	Synergism	Synergism	Dilution	No response (change < 1 μg/kg)

The response indicators are from Jarrell and Beverly^24^.

For some elements, such as Se and Mn, the application of faeces lowered their total accumulation in the forages (Fig. 2). For the application of pure faeces (i.e., with no urine), the accumulation of Se and Mn in the forages was even lower than the control with no excreta applied whatsoever, and, further, for the application of faeces + urine, the accumulation of Se and Mn in the forages was lower than when urine was applied solely (Fig. 2a). This result demonstrates that applied faeces *did* have an effect on the nutrient uptake by the plants, but for certain elements the impact was negative, at least in the short term. The lower accumulation of Se and Mn by forages after application of faeces to the soils was likely due to lower bioavailable Se and Mn in the soils, thereby decreasing Se and Mn uptake by the plants, which is reflected in the results of both the pot and field trials. The accumulation of other elements such as S and Zn in the field trial forages, for instance, was also lowered by the faecal application, but this was not observed in the pot trial (Fig. 2).

### Possible mechanisms of the nutritional response of forages to the application of different excreta

The concentration of a nutrient in a plant can be regarded as a ratio of two quantities: the accumulation of the nutrient in the plant and the plant biomass DM yield^24^, both of which can be affected by many factors such as sample harvest time, plant growth stage, and the local environment (including meteorological conditions). By considering the change in DM yield, the accumulation of a nutrient, and the nutrient concentration together (a three-vector result), we assign a *response indicator* for each nutrient-treatment-soil result (Table 5) to indicate possible interaction mechanisms by which the nutrients in the forages responded to the application of excreta. Here we consider four response indicators (Jarrell and Beverly, 1981): (1) *synergism*, in which the yield, accumulation and concentration all increased; (2) *antagonism*, in which the yield either decreased or stayed the same, but the concentration decreased; (3) *dilution*, in which the yield increased while the concentration decreased; and (4) *concentration*, in which the yield and accumulation decreased while the concentration increased. This framework was applied to the pot trial results only (Table 5) because the limited dataset for the field trial precluded identification of robust trends.

For N, Cu and Zn, the application of sheep urine, faeces, or urine + faeces led to synergism in both soils (high or low SOC). In contrast, SOC affected the response of P and S, with the urine treatment causing a dilution effect when applied to the ‘low’ SOC soil (SOC < 2% in this study), but a synergistic effect when applied to the ‘high’ SOC soil. For Mn and Se, the application of excreta appeared to cause dilution, antagonism, or no response, except for urine application to the low SOC soil, which led to a synergistic effect for Mn. A dilution or antagonistic effect on the forage concentrations of Se and Mn (Table 5) is consistent with the interpretation that the application of faeces lowers bioavailable Se and Mn in the soils, thus decreasing Se and Mn uptake by the plants (Fig. 2).

The most likely factors that could decrease Se availability in the soils treated with faeces include (1) the removal of Se from solution via precipitation, complexation, or sorption; (2) microbial activities that either compete with the forages for available Se or transform Se into less-available forms; and (3) competition of Se against other ions supplied by the application of faeces. In our previous study, we compared the soil extractable Se, S and P and soil pH across the soils treated with different excreta in the pot trial and concluded that the application of faeces to the soils could have driven greater ionic competition between SeO_3_^2−^ and PO_4_^3−^ for uptake by the perennial ryegrass, and that microbial reduction of Se (SeO_4_^2−^→SeO_3_^3−^ or SeO_3_^3−^→Se^0^) may have occurred, which also would have lowered the bioavailability of Se in the soils^25^. However, the results of the field trial in the present study provide no clear evidence of elemental competition between Se and P for uptake by forages: the accumulation of Se by the forages was lowered by the application of faeces to the field plots, but the P accumulation did not increase (Fig. 2b). Therefore, a (bio)chemical reaction that lowered the availability of Se in the soils after the application of faeces is more likely. Olson and Papworth^26^ reported decreased Se concentrations in both alfafa (*Medicago sativa*) and timothy (*Phelum pratense*) after repeated application of cattle and pig manure to soils over a five-yr period. The Se concentrations in the forages from their manured-plots were lower than those from plots without the manure application, which was attributed to microbial reduction of Se stimulated by the input of organic matter to the soils. Although, the data in that study were reported in concentrations, not accumulation in the forages, the effect of manure application on lowering the Se uptake by forages was consistent with our results.

The mechanism by which faeces application to soil decreased Mn uptake by forages remains unclear. In the pot trial, the faecal treatment had higher soil pH (pH = 6.36) compared to the untreated soils (pH = 6.17), which might have affected the availability of Mn, because the mobility of metal elements is favored by acidic environments. However, Cu and Zn have similar pH dependences on mobility to Mn, but the faecal treatment did not decrease their accumulation in forages; therefore, the impact of faeces on soil pH is unlikely to be the main cause of the decreased Mn uptake. An alternative mechanism is the microbial oxidation and biomineralization of Mn, which is widespread in soil environments^27^ and can lower bioavailable Mn in soil, thereby decreasing uptake by plants. In fact, Mn oxidation and Se reduction can be mediated by the same organism at the same time, as documented by Rosenfeld et al.^28^ in a series of experiments with two common environmental Ascomycete fungi, *Paraconiothyrium sporulosum* and *Stagonospora sp.,* both of which were observed, separately, to catalyze the reduction and partial removal of dissolved Se (IV or VI) from solution as Se(0) or organo-Se and the concomitant oxidation and removal of dissolved Mn(II) as insoluble MnO_2_ minerals. Therefore, In the present study, microbial activity driven by the faecal treatment could have transformed Mn and Se into less labile forms, via oxidation and reduction, respectively, lowering the bioavailability of these nutrients to the plants and resulting in the decreased accumulation of Mn and Se observed in the forages. However, further experiments and evidence are needed to test this inference.

### Overview and future implications

The results of this study proved that the research hypotheses were not correct. Although the different chemical forms of the supplemented minerals did not show significant impacts on the subsequent recycle of nutrients, the field trial implied that different pasture management systems could affect the nutrients in animal excreta. The application of the animal excreta did show significant impact on the accumulation of nutrients in the forages. However, contradictory to our prediction, the total input quantity of an element was not the determinative factor to its accumulation in forages. Instead, the results showed that the type of excreta (urine or faeces) nutrients were sourced from was more effective. Furthermore, the impact of different types of excreta on the elemental uptake by the forages was element-dependent. In this study, we further propose that the nutrient elements can be split into three groups based on the responses to excreta application: (1) N, Cu and Zn, (2) P and S, and (3) Mn and Se (Fig. 3, Table 5). In Group 1, N, Cu and Zn, the input of urine and/or faeces to the soil had positive, or synergistic, effect on the DM yield of perennial ryegrass, and the accumulation and concentration of these elements in the plants. Therefore, it is reasonable to expect increased N, Cu and Zn uptake by perennial ryegrass under the application of either sheep urine and/or faeces. However, the responses are not as predictable for the other two elemental groups. For the elements in Group 2, P and S, the response largely depended on the soil properties and for the elements in Group 3, Mn and Se, the application of sheep excreta, regardless of the type, tended to decrease their uptake by perennial ryegrass through either dilution or antagonism. Therefore, extra attention to the concentrations of Mn and Se in forage is needed in pastures applied with excreta.

The requirement level of Mn is between 15.05 and 22.86 mg-kg-DM^−1^ for growing lambs^12^. The concentrations of the Mn in the perennial ryegrass of the pot experiment were higher than this range for each cutting of the grass under treatments. The Mn concentration in the forages of NWFP is ca. 165 mg kg-DM^−1^ and the mean Mn concentration in UK pastures is 100 mg kg-DM^-1^, which are all higher than the typical requirement levels of sheep^2^.The antagonistic effect on Mn of applying feaces is thus not a significant issue for typical UK pastures. However, the Se concentrations in the perennial ryegrass of the pot trial were all lower than the requirement levels (between 0.16 and 0.48 mg kg-DM^−1^ for growing lambs^12^) in all cuts and of all treatments and the mean Se concentrations in the forages of NWFP and that of the UK pasture are 0.04 and 0.07 mg kg-DM^−1^, respectively^2^. Therefore the dilution and the antagonistic effects on Se concentration in forages caused by applying sheep urine and/or faeces to soils is of critical importance for UK pasture systems. To briefly conclude by building upon the broader sustainability narrative touched upon throughout the document, as environmental impact assessments are becoming increasingly complex at the farm-level, with one such example being demonstrated by Lee et al.^19^, datasets such as those generated in the present study are imperative to truly evaluate agricultural systems at a system-scale; further, such studies (i.e., pot/plot/field trials and subsequent modelling exercises) need to be carried out with more ambition in low-middle income nations where gaps in data, and more importantly, equitable food security, is more prevalent than other areas of the world.

### Supplementary Information

Supplementary Information.

## Data Availability

Sequence data that support the findings of this study is provided within the manuscript or supplementary information files. The raw data that generated in the pot trial and in the sheep experiment where the used urine and faeces were collected are available from the Rothamsted Research data repository via 10.23637/rothamsted.98883, 10.23637/rothamsted.98v24, respectively.
