# Supplementary material for: The role of ruminant urine and faeces in the recycling of nutrients by forages
Source: Sci Rep. 2024 Jul 11;14:16007. doi: 10.1038/s41598-024-66648-z (PMC11239804; doi:10.1038/s41598-024-66648-z)
Supplement: Supplementary file 1 — Supplementary Information. [file 41598_2024_66648_MOESM1_ESM.docx]

Supplementary Information

**Figure S1** Timeline of the pot experiment. The arrows in brown, pink, blue and green indicate the timings of sampling soil, sampling soil solution, applying the heavy rain event, and cutting the grass, respectively. Figure modified from Kao et al. (2023a).


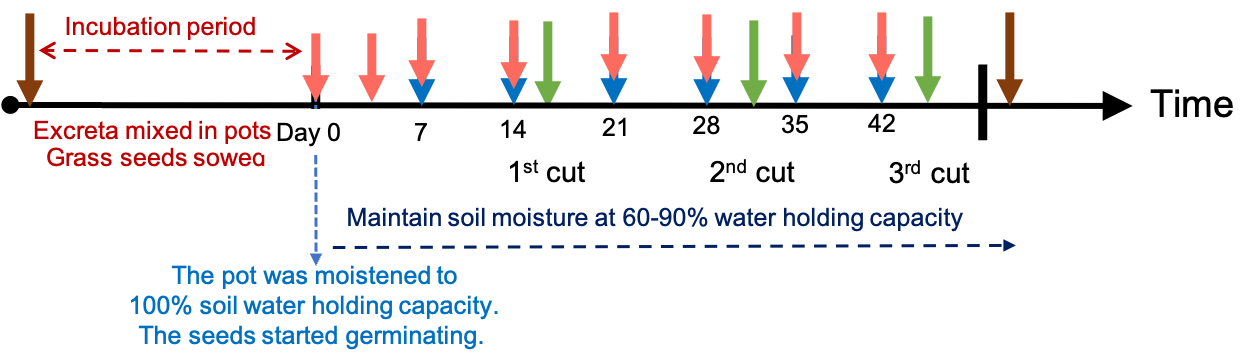


**Figure S2** Timeline of the pasture management strategies of NWFP (left) and the timeline of the current field experiment (right).


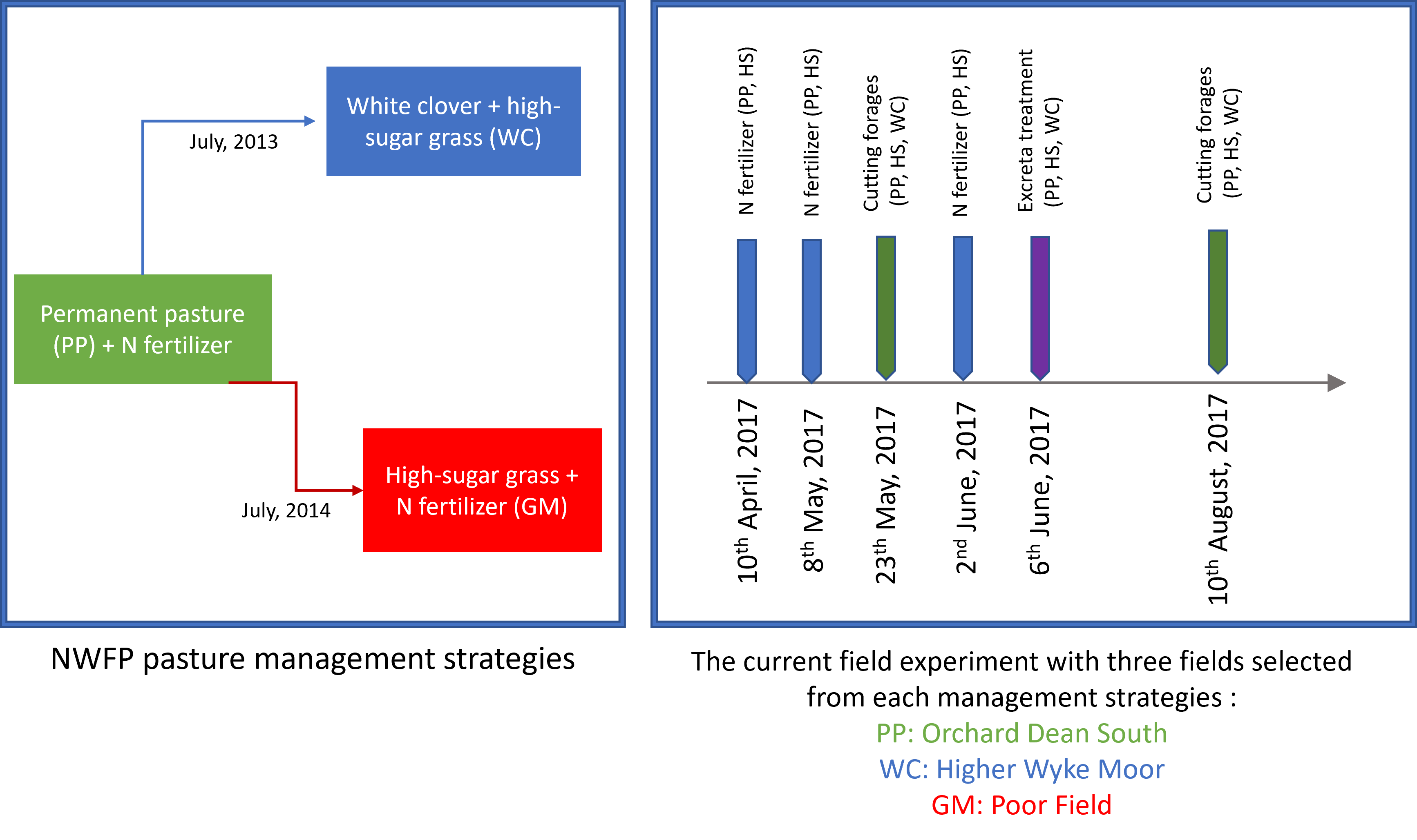


**Figure S3** The facility used for feeding the sheep and collecting the sheep urine and faeces. (a) Biocontrol system with individual silage bin of which the weight can be measured and recorded automatically. (b) The inside of an individual pen. The faeces was collected using the diaper and the urine went down through the slat and was collected in the tray shown in (a). Figure modified from Kao et al. (2023b).


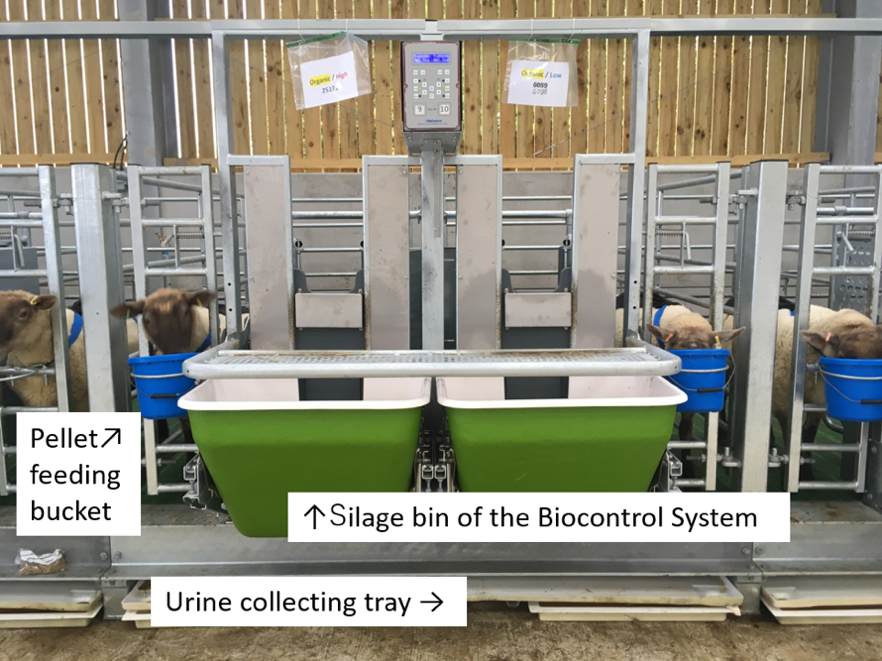

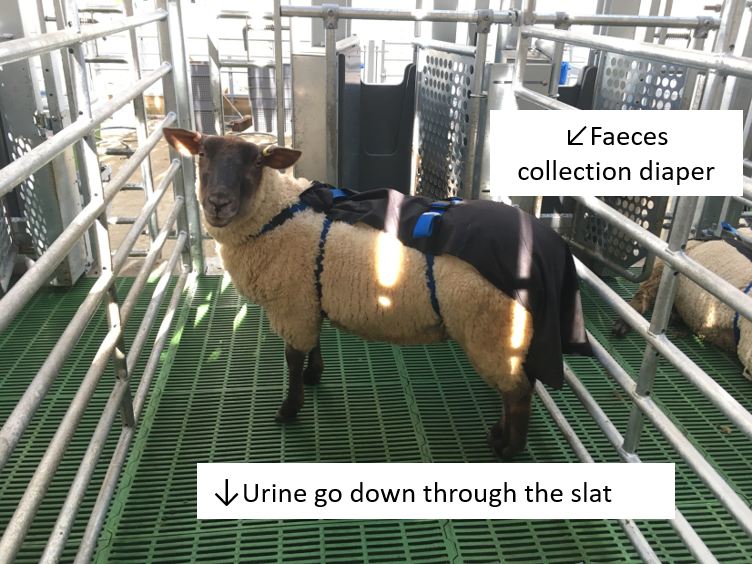


(a)

(b)

**Table S1**. The isotope mass and wavelength used and detection limit for each element in the ICP-MS and ICP-OES

| Element | ICP-MS | | ICP-OES | | |
| --- | --- | --- | --- | --- | --- |
|  | Isotope mass (amu) | Detection limit  (µg L^-1^) | Wavelength (nm) | Detection limit plant digests (mg L^-1^) | Detection limit soil digests (mg L^-1^) |
| Cd | 111 | 0.01 | - |  |  |
| Co | 59 | 0.05 | 228.616 | 0.002 | 0.002 |
| Cu | 63 | 0.03 | 327.393 | 0.005 | 0.006 |
| Fe | 57 | 0.10 | 238.204 | 0.031 | 0.695 |
| Mn | 55 | 0.02 | 257.610 | 0.001 | 0.020 |
| Mo | 95 | 0.02 | - |  |  |
| P | - |  | 213.617 | 0.256 | 0.132 |
| S | - |  | 181.975 | 0.098 | 0.670 |
| Se | 78 | 0.04 | - |  |  |
| Zn | - |  | 206.200 | 0.007 | 0.029 |
| Na | - |  | 589.592 | 0.170 | 0.149 |
| Ca | - |  | 315.887 | 0.027 | 0.283 |


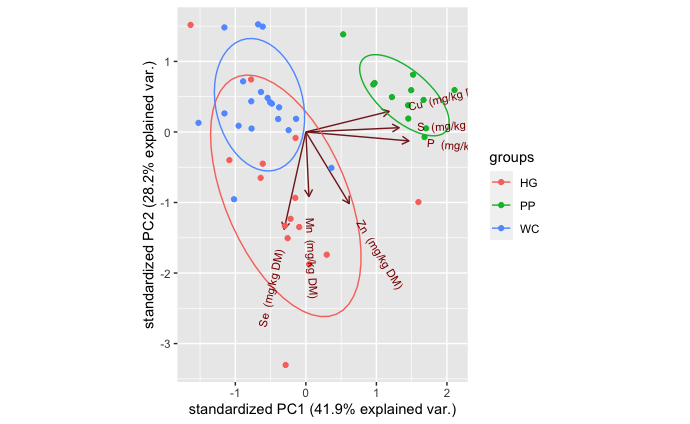


PP

WC

GM

**Figure S4** PCA analysis of soil of different fields using the concentrations of Cu, Zn, Mn, Se, S and P in the soils. The soil samples were collected in June 2016 (approximately one year before the field experiment).

**Table S2**. Nutrient concentrations in the forages before the application of cattle manure to the fields

|  | PP | WC | GM |
| --- | --- | --- | --- |
| N (w/w% in DM) | 3.08 (n=9) | 2.13 (n=11) | 2.59 (n=9) |
| P (mg kg-DM^-1^) | 3415 (n=2) | 3280 (n=2) | 3364 (n=2) |
| S (mg kg-DM^-1^) | 2760 (n=2) | 2914 (n=2) | 2383 (n=2) |
| Cu (mg kg-DM^-1^) | 6.86 (n=2) | 6.23 (n=2) | 6.22 (n=2) |
| Zn (mg kg-DM^-1^) | 23.0 (n=2) | 19.0 (n=2) | 22.3 (n=2) |
| Mn (mg kg-DM^-1^) | 98.8 (n=2) | 103 (n=2) | 71.5 (n=2) |
| Se (mg kg-DM^-1^) | 0.062 (n=2) | 0.056 (n=2) | 0.053 (n=2) |

**Table S3**. Nutrient input from the animal excreta in the pot experiment and the field trial

| Applied excreta | | N | P | S | Cu | Zn | Mn | Se |
| --- | --- | --- | --- | --- | --- | --- | --- | --- |
|  |  | Pot experiment  (Faeces from inorganic treatment: 22 g-DM pot^-1^; Faeces from organic treatment: 26 g-DM pot^-1^; Urine: 70 mL pot^-1^) | | | | | | |
|  |  | (g pot^-1^) | (mg pot^-1^) | (mg pot^-1^) | (mg pot^-1^) | (mg pot^-1^) | (mg pot^-1^) | (μg pot^-1^) |
| Faeces from the inorganic treatment | | - | 271 | 77.4 | 1.05 | 7.55 | 8.58 | 12.7 |
| Faeces from the organic treatment | | - | 316 | 96.1 | 1.24 | 8.58 | 11.1 | 15.8 |
| Urine from the inorganic treatment | | 105 | 0.245 | 82.3 | 0.003 | 0.383 | 0.008 | 2.087 |
| Urine from the organic treatment | | 116 | 0.255 | 74.3 | 0.003 | 0.376 | 0.008 | 1.553 |
| Field | Applied excreta | Field trial (Faeces: 20 kg m^-2^; Urine: 5 L m^-2^) | | | | | | |
|  |  | (g pot^-1^) | (g pot^-1^) | (g pot^-1^) | (mg pot^-1^) | (mg pot^-1^) | (g pot^-1^) | (mg pot^-1^) |
| PP | Faeces | 2524 | 757 | 267 | 2104 | 6065 | 37.5 | 12.9 |
|  | Urine | 62.1 | 276 | 62.1 | 0.267 | 3.22 | 0.001 | 1.473 |
| WC | Faeces | 2380 | 711 | 265 | 1740 | 5508 | 50.1 | 15.9 |
|  | Urine | 36.7 | 243 | 36.7 | 0.452 | 13.2 | 0.001 | 1.012 |
| GM | Faeces | 2598 | 759 | 269 | 1994 | 6352 | 31.7 | 16.1 |
|  | Urine | 32.5 | 290 | 32.5 | 0.394 | 4.48 | 0.001 | 1.441 |

**Table S4**. Soil extractable Cu, Zn and Mn in the pot experiment

| Treatments | | Extractable Cu  (mg kg^-1^) | Extractable Zn  (mg kg^-1^) | Extractable Mn  (mg kg^-1^) | Extractable NO_2_^-^+NO_3_^-^+NH_4_^+^  (g-N kg-DM^-1^ soil) | Extractable K  (mg kg^-1^) | Extractable PO_4_^3-^  (mg kg^-1^) |
| --- | --- | --- | --- | --- | --- | --- | --- |
|  |  | (mean ± SE)† | | | | | |
| Soil of low organic carbon | Untreated | 0.00±0.001 | 0.33±0.004 | 14.2±0.28 | 8.74±0.752 | 277±1.4 | 29.5±0.47 |
|  | Faeces from inorganic treatment | 0.04±0.004 | 0.11±0.004 | 6.93±0.239 | 39.4±1.78 | 385±0.7 | 63.9±1.85 |
|  | Faeces from organic treatment | 0.07±0.006 | 0.18±0.008 | 24.9±0.56 | 22.2±2.31 | 377±3.0 | 89.9±1.84 |
|  | Urine from inorganic treatment | 0.03±0.001 | 0.02±0.004 | 4.11±0.058 | 268±25.3 | 964±3.9 | 32.3±0.14 |
|  | Urine from organic treatment | 0.02±0.001^i^ | 0.02±0.001 | 3.47±0.042 | 287±11.0 | 869±2.3 | 31.9±0.54 |
|  | Urine and faeces from inorganic treatment | 0.08±0.001 | 0.04±0.001 | 5.08±0.014 | 282±6.79 | 1070±9.0 | 73.3±2.27 |
|  | Urine and faeces from organic treatment | 0.05±0.002 | 0.06±0.001 | 9.88±0.056 | 313±23.5 | 936±7.4 | 66.8±1.66 |
| Soil of high organic carbon | Untreated | 0.02±0.001 | 1.39±0.005 | 12.0±0.05 | 15.9±2.82 | 147±0.4 | 64.8±0.79 |
|  | Faeces from inorganic treatment | 0.05±0.001 | 0.63±0.009 | 3.84±0.018 | 62.3±4.89 | 228±1.1 | 95.0±4.03 |
|  | Faeces from organic treatment | 0.06±0.001 | 0.69±0.005 | 4.13±0.124 | 65.0±5.02 | 208±3.1 | 117±3.3 |
|  | Urine from inorganic treatment | 0.02±0.001 | 0.21±0.002 | 0.65±0.010 | 349±14.8 | 807±4.1 | 60.9±1.79 |
|  | Urine from organic treatment | 0.03±0.000 | 0.18±0.002 | 0.68±0.003 | 299±6.0 | 757±4.1 | 64.9±1.15 |
|  | Urine and faeces from inorganic treatment | 0.08±0.001 | 0.18±0.003 | 1.07±0.024 | 358±12.9 | 873±1.0 | 107±4.7 |
|  | Urine and faeces from organic treatment | 0.10±0.007 | 0.15±0.005 | 1.05±0.090 | 377±10.6 | 808±7.2 | 123±4.7 |
